# Developmental changes of sleep spindles and their impact on sleep‐dependent memory consolidation and general cognitive abilities: A longitudinal approach

**DOI:** 10.1111/desc.12706

**Published:** 2018-09-05

**Authors:** Michael Hahn, Ann‐Kathrin Joechner, Judith Roell, Manuel Schabus, Dominik PJ Heib, Georg Gruber, Philippe Peigneux, Kerstin Hoedlmoser

**Affiliations:** ^1^ Laboratory for Sleep, Cognition and Consciousness Research Department of Psychology Centre for Cognitive Neuroscience University of Salzburg Salzburg Austria; ^2^ Department of Psychiatry and Psychotherapy Medical University of Vienna Vienna Austria; ^3^ The Siesta Group Vienna Austria; ^4^ UR2NF – Neuropsychology and Functional Neuroimaging Research Unit affiliated at CRCN – Centre de Recherches en Cognition et Neurosciences and UNI – ULB Neurosciences Institute Université Libre de Bruxelles Bruxelles Belgium

## Abstract

Sleep spindles are related to sleep‐dependent memory consolidation and general cognitive abilities. However, they undergo drastic maturational changes during adolescence. Here we used a longitudinal approach (across 7 years) to explore whether developmental changes in sleep spindle density can explain individual differences in sleep‐dependent memory consolidation and general cognitive abilities. Ambulatory polysomnography was recorded during four nights in 34 healthy subjects (24 female) with two nights (baseline and experimental) at initial recording (age range 8–11 years) and two nights at follow‐up recording (age range 14–18 years). For declarative learning, participants encoded word pairs with a subsequent recall before and after sleep. General cognitive abilities were measured by the Wechsler Intelligence Scale. Higher slow (11–13 Hz) than fast (13–15 Hz) spindle density at frontal, central, and parietal sites during initial recordings, followed by a shift to higher fast than slow spindle density at central and parietal sites during follow‐up recordings, suggest that mature spindle topography develops throughout adolescence. Fast spindle density increases from baseline to experimental night were positively related to sleep‐dependent memory consolidation. In addition, we found that the development of fast spindles predicted the improvement in memory consolidation across the two longitudinal measurements, a finding that underlines a crucial role for mature fast spindles for sleep‐dependent memory consolidation. Furthermore, slow spindle changes across adolescence were related to general cognitive abilities, a relationship that could indicate the maturation of frontal networks relevant for efficient cognitive processing. A video abstract of this article can be viewed at: https://www.youtube.com/watch?v=7NXJzm8HbIw and https://www.youtube.com/watch?v=iuMQY1OIJ0s

**RESEARCH HIGHLIGHTS**

- Using a longitudinal study design, we investigated the developmental changes of sleep spindles across adolescence and their relation to memory consolidation and general cognitive abilities.
- Mature spindle topography developed between initial (8–11 years) and follow‐up recordings (14–18 years).
- Fast spindle density increases were associated with sleep‐dependent word pair memory consolidation.
- Fast spindle development predicted the difference in memory consolidation between initial and follow‐up recordings, whereas slow spindle development correlated with cognitive abilities.

## 1 INTRODUCTION

Decreases in total sleep time, sleep efficiency and slow‐wave sleep across the lifespan are well known (Ohayon, Carskadon, Guilleminault, & Vitiello, 2004). While these decreases follow a marginal trend from adulthood to old age, the most drastic changes in sleep architecture occur during adolescence starting with the onset of puberty between 9 and 12 years of age (Crone & Dahl, 2012; Ohayon et al., 2004; Tarokh, Saletin, & Carskadon, 2016). However, changes in sleep during adolescence are not only present at the macroscopic level of sleep architecture but also at the level of cortical oscillations unique to the sleeping brain, namely sleep spindles (Campbell & Feinberg, 2016; Nicolas, Petit, Rompre, & Montplaisir, 2001; Purcell et al., 2017; Scholle, Zwacka, & Scholle, 2007; Shinomiya, Nagata, Takahashi, & Masumura, 1999).

Sleep spindles (11–15 Hz), the most prominent feature of non‐rapid eye movement sleep 2 (NREM2), originate in the thalamus via an interplay between reticular and thalamo‐cortical neurons (Steriade, Deschenes, Domich, & Mulle, 1985). Based on frequency and topography, sleep spindles can be categorized into frontal slow spindles (11–13 Hz) and centro‐parietal fast spindles (13–15 Hz) with distinct generators (Anderer et al., 2001; Schabus et al., 2007; Zeitlhofer et al., 1997). Both spindle types share common hemodynamic thalamic activation, but while slow spindles show sources in the frontal cortex and are associated with activation in the superior frontal gyrus, fast spindles show sources in the precuneus and are associated with activation in memory‐relevant areas like mesial frontal area, sensorimotor area and hippocampus (Anderer et al., 2001; Schabus et al., 2007). At the network level, it has been suggested that slow spindles are related to cortico‐cortical couplings, whereas fast spindles are related to thalamo‐cortical couplings (Doran, 2003). Although the literature has not reached a consensus on how to exactly differentiate between slow and fast spindles, there is strong evidence for the existence of these two distinct spindle types (Cox, Schapiro, Manoach, & Stickgold, 2017; Schabus et al., 2007; Zeitlhofer et al., 1997).

The notion of two different sleep spindle types is further corroborated by their developmental trajectory. Overall sleep spindle density (number of sleep spindles per time epoch) increases and reaches a maximum in early adulthood, a development that is followed by a slower decrease across the whole lifespan (Nicolas et al., 2001; Purcell et al., 2017; Scholle et al., 2007). Spindle frequency increases from childhood to adolescence; however, slow spindle frequency plateaus earlier than fast spindle frequency (Purcell et al., 2017; Shinomiya et al., 1999). Supporting these findings, a longitudinal study showed that the spectral peak of sleep spindles (11–15 Hz) increases from childhood to adolescence. Yet, slow spindle power (11–12.8 Hz) decreased, whereas faster spindle power (13.4–14.4 Hz) increased (Campbell & Feinberg, 2016). Taken together, this indicates different developmental trajectories of slow and fast spindles.

Like sleep spindles, the brain undergoes continuous changes during maturation. Synaptogenesis, myelination and apoptosis proceed from infancy through adolescence, processes that are accompanied by a strong cortical thinning at the beginning of puberty (Huttenlocher & Dabholkar, 1997; Tau & Peterson, 2010). These processes are thought to be essential for the development of functional neural networks. As sleep spindles are related to synaptic plasticity through long‐term potentiation and white matter diffusion, it is likely that they could function as a marker of brain maturation (Piantoni et al., 2013; Rosanova & Ulrich, 2005; Shinomiya et al., 1999).

Besides long‐term potentiation, sleep spindles are temporally linked with hippocampal sharp‐wave ripples representing memory reactivation which is associated with the information transfer between the hippocampus and neocortex (Buzsaki, 1996; Clemens et al., 2011; Gais et al., 2007). With memory reactivation and long‐term potentiation being key processes of learning and memory formation, sleep spindles have received extensive attention in sleep‐dependent memory consolidation research.

In adults it has been shown that during a night following a declarative memory task compared to a night following a non‐learning control task, spindle density increased, and that spindle density was related to memory performance before and after sleep (Gais, Molle, Helms, & Born, 2002). Furthermore, only participants with increased spindle activity (i.e. a measure combining spindle duration and amplitude) in the experimental night compared to a non‐learning control night showed a boost in declarative memory performance after sleep (Schabus et al., 2004). In particular, learning‐related increases of central fast spindle activity were crucial for sleep‐dependent memory consolidation (Schabus et al., 2008).

With regard to pediatric populations, school‐aged children (6–12 years) improved their performance on word pair, novel non‐word and object location knowledge tasks after sleep (Backhaus, Hoecksfeld, Born, Hohagen, & Junghanns, 2008; Brown, Weighall, Henderson, & Gaskell, 2012; Wilhelm, Diekelmann, & Born, 2008). Sleep‐dependent memory consolidation of 6–8‐year‐olds was comparable to adults but unrelated to any sleep parameters, whereas sleep‐dependent memory consolidation of 9–12‐year‐olds was related to higher percentages of NREM sleep (Backhaus et al., 2008; Wilhelm et al., 2008).

Considering sleep spindles, results in adult populations suggest that spindles are crucial for sleep‐dependent memory consolidation. However, evidence for the significance of sleep spindles for memory consolidation in children is still lacking, with only a few studies focusing on this relationship. In one of our studies, we were able to show that slow spindle activity was not related to sleep‐dependent memory consolidation, but was linked to higher initial memory performance in 8–11‐year‐old children (Hoedlmoser et al., 2014). On the other hand, central spindle density (11.84 Hz) was positively associated with sleep‐dependent memory consolidation after a nap in a spatial memory task, but negatively associated with immediate recall performance in 3–6‐year‐old children (Kurdziel, Duclos, & Spencer, 2013). However, given the changes during puberty described above, differences in spindle functionality in terms of age are not surprising.

Sleep spindles show high individual stability and are strongly defined by genetics, although they are subject to change across maturation (De Gennaro et al., 2008; Landolt, 2011). Likewise, intelligence is considered to be a trait, that is, it remains stable across aging. Links between spindle characteristics and general cognitive abilities have been repeatedly reported in adults (Fogel & Smith, 2011). For example, high frontal fast spindle density was found to predict higher general cognitive abilities, a relationship that has also been found for slow and fast spindle activity (Bodizs et al., 2005; Schabus et al., 2006).

To date, the relationship between sleep spindles and intelligence is less understood in children than in adults. Nevertheless, sleep spindles could provide valuable information about the development of cognitive functions. Higher power in the spindle frequency band (12–15 Hz; sigma frequency) was positively related to general and fluid intelligence, but a higher frequency peak was negatively related to general intelligence in 9–11‐year‐old children (Geiger et al., 2011). Similarly lower spindle frequency was related to better reasoning and working memory in school‐aged children, a result that is in contrast to studies of adult populations (Chatburn et al., 2013; Gruber et al., 2013). In both adolescents (15–22 years) and children (4–8 years), fast spindle amplitude was positively correlated with fluid intelligence (Bodizs, Gombos, Ujma, & Kovacs, 2014; Ujma, Sandor, Szakadat, Gombos, & Bodizs, 2016). Intelligence scores are generally normalized based on age and therefore do not change across aging. However, cognitive performance measured by raw scores markedly increases from childhood to adulthood (Ujma et al., 2016). As sleep spindles also change profoundly during that time, we hypothesize that they could provide a biological model for the development of cognitive abilities.

Taken together, growing evidence suggests that sleep spindles play an equally prominent role in children and adults for sleep‐dependent declarative memory consolidation and general cognitive abilities. However, the few studies that investigated these relationships differed in age ranges, utilized learning tasks, the investigated spindle parameters and frequency definitions of slow and fast spindles. Therefore, the picture of sleep spindles in children and adolescents is more heterogeneous than in adults.

We reported earlier that when discriminating between slow and fast spindles, the spindle peak is restricted to the slow spindle frequency in 8–11‐year‐old children. Furthermore, memory performance decreased after a sleep retention interval, with no relationship to slow spindle activity. However, slow spindle activity predicted higher initial memory performance and higher general cognitive abilities (Hoedlmoser et al., 2014).

Our longitudinal study aimed to answer whether developmental changes in sleep spindles are involved in the development of individual differences in memory consolidation and general cognitive abilities. At two developmental stages: (i) during childhood and (ii) during adolescence, participants learned word pairs and were tested before and after full‐night polysomnography (PSG). Based on the aforementioned literature, we hypothesized that participants would improve memory performance and sleep‐dependent memory consolidation from childhood to adolescence and show an increased appearance of fast sleep spindles. Further, we expected that subjects with better sleep‐dependent memory consolidation would show increased fast sleep spindle density compared to participants with worse memory consolidation. Likewise, the development of fast sleep spindles was hypothesized to be related to the changes in sleep‐dependent memory consolidation from childhood to adolescence. Furthermore, as sleep spindles are known to be related to cognitive abilities, developmental spindle changes were expected to be associated with general cognitive abilities.

## 2 METHODS

### 2.1 Subjects

Thirty‐six healthy subjects (24 females) were recruited from public elementary schools and originate from a previously investigated dataset (Hoedlmoser et al., 2014). They were 8–11 years old at initial data acquisition (INI) and 14–18 years old at follow‐up data acquisition (FUP). Due to technical difficulties in two PSG nights, we excluded two participants from further analyses and therefore report the data of 34 subjects (24 females; INI mean age: 9.44 ± 0.79, FUP mean age: 15.97 ± 0.87). The stage of pubertal development was assessed by the pubertal development scale (Carskadon & Acebo, 1993). The majority of participants were rated pre‐pubertal at INI (68%) and late to post pubertal at FUP (91%; cf. supplemental material: Figure S1). Before entering the study, participants were screened for possible exclusion criteria, such as sleep and mental disorders, respiration problems and medication influencing sleep or cognitive abilities. Sleep quality was assessed using the Children's Sleep Habits Questionnaire (Owens, Spirito, & McGuinn, 2000) at INI and the Pittsburg Sleep Quality Index (Buysse, Reynolds, Monk, Berman, & Kupfer, 1989) at FUP. To measure general cognitive abilities, we used the German version of the Wechsler Intelligence Scale for Children at INI (Petermann & Petermann, 2007) and the Wechsler Adult Intelligence Scale at FUP (Wechsler, 1997). We restricted our assessment to the subtests Vocabulary, Matrix Reasoning and Block Design for a prorated intelligence quotient, to reduce the duration of the already very extensive screening procedure. Participants and their parents were informed about the course and aim of the study and gave their written informed consent. Participants received a gift at INI (Professor Globus, Leap Frog Enterprises, Inc., California, USA) and €100 at FUP. The study was conducted in accordance with the Declaration of Helsinki.

### 2.2 Procedure

**Figure 1:**
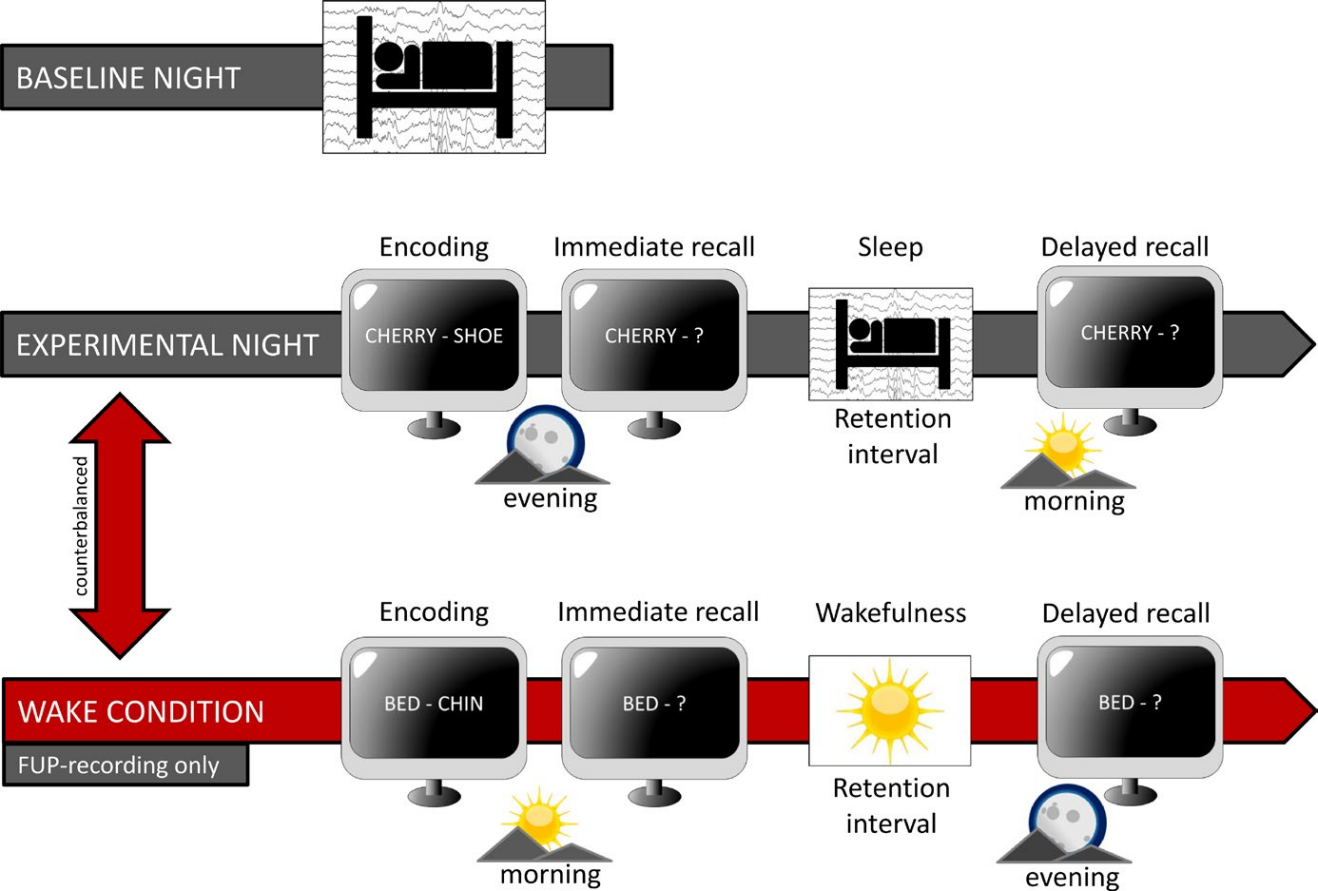
Study design. Ambulatory PSG recordings were conducted at the participants’ homes. During both nights, participants stayed in bed for 10 hours at initial and 8 hours at follow‐up recordings. At the experimental night, word pair encoding was performed in the evening with subsequent recall one hour before sleep and a delayed recall one hour after sleep. This resulted in a 12 hour retention interval at initial and a 10 hour retention interval at follow‐up recordings. The counterbalanced wake condition was only conducted for follow‐up recordings with encoding and immediate recall in the morning followed by 10 hours of wakefulness prior to the delayed recall in the evening

At both data acquisition periods (Figure 1), ambulatory PSG recordings were conducted at the participants’ homes in their habitual sleeping environment. Environmental influences that could disturb sleep (e.g. light, noise, temperature) were controlled. In addition, participants slept alone in their room, without the presence of parents, siblings or pets, to minimize disturbances during the night. To check for compliance with maintaining a regular sleep schedule, participants wore a wrist actigraph (Cambridge Neurotechnology Actiwatch ©, Cambridge, UK) and completed a sleep log (Saletu, Wessely, Grünberger, & Schultes, 1987). Sleep was recorded for two nights at each data recording phase. At the baseline night, participants slept without any interventions according to their regular sleep time. In cases of first night effects, participants were excluded from analyses. At the experimental night, participants performed a declarative learning task before and after sleep. Encoding and the immediate recall were completed 1 hour (h) before lights off, whereas the delayed recall was performed 1 h after waking in the morning. Time in bed was held constant between the two nights. However, due to the different school schedules of the participants at INI and FUP, adaptations were made to the study design. At INI, the two PSG recordings were obtained on school days and separated by seven days. Time in bed was fixed for 10 h resulting in lights off from 8.30 p.m. to 6.30 a.m. (for a more detailed description, see Hoedlmoser et al., 2014). At FUP, lights off was set between 11 p.m. and 7 a.m. for a total of 8 h of time in bed. Data recordings were only conducted on weekends, with the baseline night from Friday to Saturday and the experimental night from Saturday to Sunday. Participants were instructed to maintain their normal school day sleep rhythm. At FUP a counterbalanced wake condition one week before or after the PSG recordings was added. During wake condition encoding and the immediate recall of a new set of word pairs took place at 8.00 a.m. The delayed recall in the evening was separated by a 10 h retention interval, similar to the sleep condition but with the participants staying awake.

### 2.3 Declarative learning task

Participants had to encode non‐associated, not semantically related word pairs (e.g. ‘cherry–shoe’) at INI and FUP. Word pairs were presented on a laptop screen in white font on a black background. During encoding, the set of word pairs was shown twice in randomized order. To control for different mnemonic strategies, participants were instructed to imagine a visual connection between the two words presented. The immediate cued recall session was performed after a 10 minute (min) break and 1 h before lights off. In this session only the first word (cue) of the pair was presented (‘cherry–?’). Participants had to press the left mouse button and name the second word of the pair. Word pair cues were presented in randomized order. Participants were instructed to answer as fast and as accurately as possible. They did not receive any feedback on their performance. The delayed recall session was performed 1 h after lights on, resulting in a 12 h retention interval containing sleep at INI and a 10 h retention interval either containing sleep or wakefulness depending on the condition (wake, sleep) at FUP.

At INI, participants encoded 50 word pairs, which were presented for 5 seconds (s), followed by a fixation cross for 3 s during encoding. This encoding session lasted for 20 min. In the recall sessions, the first word of the pair was shown for a maximum time of 10 s followed by a fixation cross for 1.5 s. The maximal duration of each recall session was 9.5 min.

In order to prevent ceiling effects of performance at FUP, we increased the number of word pairs and adjusted the timing. Therefore, two independent 80 word pair lists were created for the sleep and the wake conditions at FUP. Word pairs were presented for 6.5 s, followed by a fixation cross for 3.5 s (Schabus et al., 2004). The timing of the word pairs during encoding and recall was equal. The encoding session lasted 26 min and each recall session maximal 13 min.

Declarative memory performance was defined according to Schabus et al. (2004) and Hoedlmoser et al. (2014): The recall score consisted of (1) number of correctly recalled word pairs and (2) and semantic (unambiguous) correct word pairs (e.g. ‘boot’ or ‘sandal’ instead of ‘shoe’). Semantically correct pairs were weighted by 0.5. Recall performance was expressed as percentage (recall score/total count of word pairs * 100). We used this performance measure for optimal comparability to our earlier study (Hoedlmoser et al., 2014). However, analyzing only correctly recalled word pairs would not have changed our results.

### 2.4 Sleep recordings

PSG was recorded with a portable amplifier system (Alphatrace, Becker Meditec, Germany) at a sampling rate of 512 Hz. The EEG signal was recorded using gold‐plated electrodes (Grass Technologies, Astro‐Med GmbH, Germany) on channels F3, Fz, F4, C3, C4, P3, Pz, P4, O1 and O2 and online referenced against a common reference at Cz. Cz was retrieved after re‐referencing offline to averaged channels A1 and A2 at the mastoids. Four EOG channels were used: two horizontal channels, one placed above the right outer canthus, the other below the left outer canthus and two vertical channels above and below the right eye. EMG was derived from two electrodes over the left and right musculus mentalis. Sleep was automatically staged (Somnolyzer 24 × 7, Koninklijke Philips N.V.; Eindhoven, The Netherlands) and visually controlled by an expert scorer according to the American Academy of Sleep Medicine criteria (AASM; Iber, Ancoli‐Israel, Chesson, & Quan, 2007).

Sleep spindles were detected independently during NREM2 for frontal (F3, Fz, F4), central (C3, Cz, C4), and parietal (P3, Pz, P4) derivations. The automatic spindle detection (ASK analyzer, The Siesta Group, Vienna, Austria) was twofold: In the first step, ‘possible’ spindle events were detected by the band‐pass method (Schimicek, Zeitlhofer, Anderer, & Saletu, 1994) with the following criteria: (1) Band‐pass filter 11–16 Hz, (2) amplitude > 12 μV, (3) duration 300 – 2000 ms. In the second step, these possible spindle events detected by this low specificity but high sensitivity method were further evaluated in order to increase specificity. From all ‘possible’ spindle episode candidates, ‘certain’ spindle episodes were identified by linear discriminant analysis (LDA) trained on visually scored spindles. The LDA uses the five log‐transformed features (spindle duration and mean amplitudes in four frequency bands: spindle, theta, alpha, and fast beta) of ‘possible’ spindles. For all our analyses we only used spindle events with a discriminant score > 1.7. This corresponds to a specificity of 98%, which is similar to visual scorers (for more details, see Anderer et al., 2005). Furthermore, we visually inspected the spindles identified by the algorithm to ensure valid detections (for exemplary spindle detections, see Figure S2). Spindle density (mean number per minute of NREM2 sleep) was calculated for each electrode and averaged separately for frontal (F3, Fz, F4), central (C3, Cz, C4), and parietal (P3, Pz, P4) derivations. The spindle frequency was determined in the time domain by period amplitude analysis of the band‐pass filtered signal. Based on the spindle frequency we further differentiated between slow (11–13 Hz) and fast (13–15 Hz) spindles.

### 2.5 Statistical analyses

Analyses were mainly based on repeated measure and mixed analyses of variance (ANOVA). For correlations, Spearman rho coefficients (r_s_), and partial correlation coefficients (r_p_) are reported. Partial eta squared (p.eta²) and Cohen's *d* (*d*) are reported for effect sizes. Degrees of freedom are Greenhouse‐Geisser corrected. Post‐hoc tests are Bonferroni corrected. Post‐hoc tests that did not survive correction are marked by a ‘†’.

Overnight memory change was calculated by subtracting immediate recall performance from delayed recall performance. Changes in spindle density from baseline to experimental night were calculated by subtracting baseline values from experimental night values. Developmental changes of sleep spindles were computed by subtracting values of INI from FUP. In a similar way, development of overnight memory change between INI and FUP was computed [(delayed_FUP_ − immediate_FUP_) − (delayed_INI_ − immediate_INI_)]. It should be noted that this value does not indicate a successful overnight memory gain, but states whether the subject forgot less or remembered more words after the sleep retention interval at FUP relative to INI.

## 3 RESULTS

### 3.1 Behavioral data: declarative memory

Recall performance between INI and FUP was positively correlated (immediate: r_s_(34) = 0.48, *p* = 0.004, delayed: r_s_(34) = 0.44, *p* = 0.009). In contrast, overnight memory change was not correlated between the INI and FUP (r_s_(34) = −0.01, *p* = 0.978). At both recordings, overnight memory change was independent of the intelligence score (INI: r_s_(34) = 0.15, *p* = 0.403, FUP: r_s_(34) = −0.001, *p* = 0.999). Intelligence scores correlated with immediate recall performance only at FUP (INI: r_s_(34) = 0.13, *p* = 0.457, FUP: r_s_(34) = 0.54, *p* = 0.001). There were no gender differences, either in recall performance (INI: *t*(32) = 0.22, *p* = 0.83, *d* = 0.08; FUP: *t*(32) = 0.29, *p* = 0.77, *d* = 0.11) or in overnight memory change (INI: *t*(32) = 0.80, *p* = 0.43, *d* = 0.30; FUP: *t*(32) = 0.53, *p* = 0.60, *d* = 0.20).

**Figure 2:**
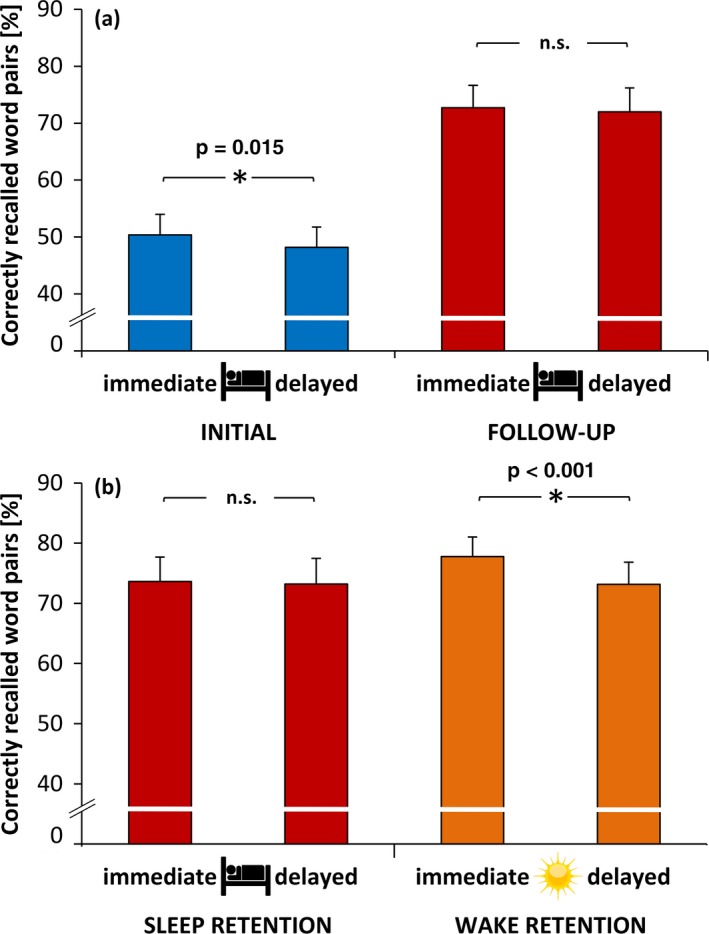
(a) Means and standard error of correctly recalled word pairs compared between initial and follow‐up recordings before and after a sleep retention interval. Memory performance was higher at follow‐up recordings. At initial recordings, word pair memory decreased after the sleep interval (p = 0.015) but remained stable at follow‐up recordings. (b) Comparison of word pair memory between a sleep and a wake retention interval at follow‐up recordings. Memory did not change after the sleep retention interval but decreased after the wake retention interval (p <0.001)

A repeated measure ANOVA with the factors Maturation (INI, FUP) and Recall Time (immediate, delayed) showed that, overall, participants recalled a higher percentage of word pairs at FUP than at INI (Figure 2a; *F*(1, 33) = 41.33, *p* < 0.001, p.eta² = 0.56). At both recording phases, recall performance was higher before than after sleep (*F*(1, 33) = 7.69, *p* = 0.009, p.eta² = 0.19). In addition, there was a trend for an interaction between Maturation and Recall Time (*F*(1, 33) = 2.75, *p* = 0.106, p.eta² = 0.08). Planned contrasts showed that participants had a significant overnight memory decrease at INI (*t*(33) = 2.55, *p* = 0.015, *d* = 0.13), whereas memory remained stable at FUP (*t*(33) = 1.1, *p* = 0.261, *d* = 0.03). To sum up, participants increased their recall performance from childhood to adolescence and benefited more from a sleep retention interval during adolescence.

In a second step, we analyzed whether sleep has a positive influence on memory consolidation as opposed to a wake retention interval (Figure 2b; only for FUP, as no wake condition was conducted at INI). Because two subjects did not participate in the wake condition, we present the data for 32 participants only. A repeated measure ANOVA with the factors Recall Time (immediate, delayed) and Retention Condition (sleep, wake) showed that, overall, participants did not differ between immediate and delayed recall (*F*(1, 31) = 0.53, *p* = 0.472, p.eta² = 0.02). However, they recalled more word pairs in the wake condition than in the sleep condition (*F*(1, 31) = 16.69, *p* < 0.001, p.eta² = 0.35). In addition, we found an interaction between Recall Time and Retention Condition (*F*(1, 31) = 34.47, *p* < 0.001, p.eta² = 0.53). Planned contrasts showed that participants did not differ in immediate recall performance between sleep and wake condition (*t*(31) = −1.49, *p* = 0.145, *d* = 0.19). However, participants decreased their recall performance from immediate to delayed recall in the wake condition (*t*(31) = 5.77, *p* < 0.001, *d* = 0.20), whereas their performance remained stable in the sleep condition (*t*(31) = 0.73, *p* = 0.47, *d* = 0.02). In summary, these results indicate that as opposed to a wake retention interval, a sleep retention interval was beneficial for memory consolidation during adolescence.

### 3.2 Sleep architecture

Sleep architecture did not differ between the baseline and experimental nights in each session (all *p* > 0.05). Therefore, we could rule out a first night effect. However, due to longer time in bed in the INI compared to FUP session, between‐session differences were found (for a summary see Table 1).

**Table 1:** Sleep architecture for initial and follow‐up recordings

	Initial (INI)		Follow‐up (FUP)		*p*‐values			
	Baseline	Experimental	Baseline	Experimental	Base_INI_*Exp_INI_	Base_FUP_*Exp_FUP_	Base_INI_*Base_FUP_	Exp_INI_*ExP_FUP_
TIB(min)	584.60 ± 41.50	588.62 ± 38.24	494.46 ± 58.86	494.1 ± 47.77	0.55	0.97	< 0.001*	< 0.001*
SOL to N2(min)	19.81 ± 10.47	23.84 ± 16.17	19.96 ± 20.98	16.81 ± 13.42	0.15	0.44	0.97	0.05
WASO(min)	13.26 ± 14.16	8.96 ± 9.33	15.88 ± 18.31	10.03 ± 6.79	0.07	0.05	0.49	0.53
TST(min)	555.47 ± 43.70	562.09 ± 35.04	471.82 ± 62.66	474.82 ± 44.73	0.29	0.77	< 0.001*	< 0.001*
EFF(%)	95.07 ± 3.00	95.48 ± 2.69	95.39 ± 4.44	96.25 ± 2.24	0.49	0.27	0.70	0.16
N1(%)	2.92 ± 2.57	2.62 ± 1.78	9.14 ± 4.19	8.80 ± 4.68	0.38	0.66	< 0.001*	< 0.001*
N2(%)	44.64 ± 7.45	44.09 ± 7.99	41.65 ± 7.07	41.17 ± 6.66	0.61	0.45	0.03*	0.03*
N3(%)	26.41 ± 9.72	25.63 ± 9.49	25.01 ± 9.87	24.64 ± 9.48	0.22	0.64	0.47	0.59
R(%)	26.02 ± 6.13	27.76 ± 7.66	24.20 ± 5.53	25.38 ± 5.14	0.06	0.18	0.17	0.11

Note

TIB, time in bed; SOL to N2, sleep onset latency; WASO, wake after sleep onset; TST, total sleep time; EFF, sleep efficiency.

Asterisks indicate a significant difference at the 0.05 level.

### 3.3 Sleep spindle maturation

**Figure 3:**
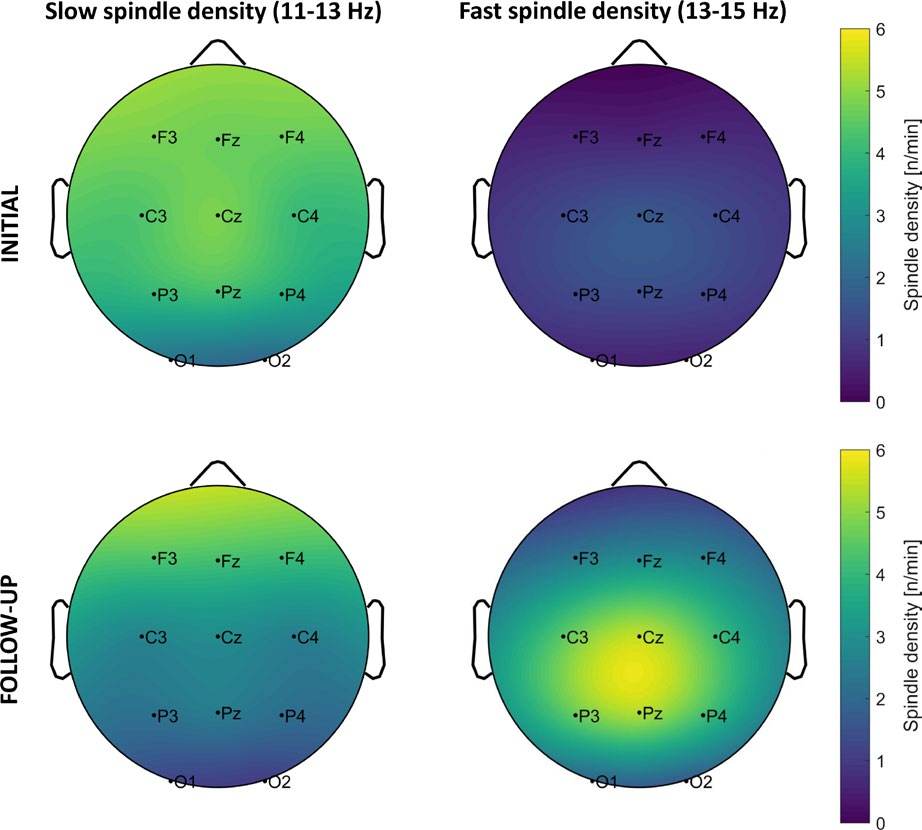
Topographical plots for mean spindle density at each electrode separated by slow (left column) and fast spindle density (right column) at initial (upper row) and follow‐up recordings (lower row) during experimental nights. Bright colours indicate higher spindle density. Slow spindles were dominant during initial recordings. At follow‐up, slow spindles only remained dominant at frontal derivations, whereas fast spindles became dominant at centro‐parietal derivations. Note that due to the extrapolation of topographical plots, colours that go beyond the measured electrodes do not contain any information

Result patterns for sleep spindle density were the same for baseline and experimental nights. Therefore, only statistics of the experimental night are reported in the main text (Figure 3; for results of the baseline night, see Table S1 and Figure S3). A repeated measure ANOVA with the factors Maturation (INI, FUP), Electrode (frontal, central, parietal) and Spindle Type (slow, fast) showed that participants had higher overall spindle density at FUP than at INI (*F*(1, 33) = 35.63, *p* < 0.001, p.eta² = 0.52). However, spindle density differed in relation to Maturation, Electrode and Spindle Type (*F*(1.29, 42.43) = 39.21, *p* < 0.001, p.eta² = 0.54). Post‐hoc tests (Bonferroni corrected: 0.05/12 = 0.0042) showed that participants had higher slow than fast spindle density at all electrode sites at INI (*t*(33)_frontal_ = 14.14, *p* < 0.001, *d* = 2.63; *t*(33)_central_ = 9.62, *p* < 0.001, *d* = 2.09; *t*(66)_parietal_ = *p* < 0.001, *d* = 1.80). At FUP, participants showed higher slow than fast spindle density at frontal sites (*t*(33) = 3.81, *p* = 0.001, *d* = 0.96), whereas fast spindle density was higher at central (*t*(33) = −3.19, *p* = 0.003, *d* = 0.95), and parietal sites (*t*(33) = −3.48, *p* = 0.001, *d* = 1.09). Except at frontal sites (*t*(33) = 1.62, *p* = 0.116, *d* = 0.20), slow spindle density decreased from INI to FUP (*t*(33)_central_ = 5.52, *p* < 0.001, *d* = 1.05; *t*(33)_parietal_ = 5.76, *p* < 0.001, *d* = 1.04). In contrast, fast spindle density increased at all electrode sites from INI to FUP (*t*(33)_frontal_ = −7.40, *p* < 0.001, *d* = −0.98; *t*(33)_central_ = −10.13, *p* < 0.001, *d* = −1.31; *t*(33)_parietal_ = −10.05, *p* < 0.001, *d* = −.1.40). Taken together, these results show that while slow spindle density dominated during childhood, fast spindle density matured throughout adolescence.

Previous literature suggests that spindles also change in frequency during development, which makes it challenging to interpret findings solely regarding the density of slow and fast spindles. Therefore, we further investigated spindle frequency in addition to spindle density and found an overall increase of frequency at all electrode sites (see Table S2 and Figure S4 for these supplemental results).

### 3.4 Sleep spindles and declarative memory consolidation

Because central and parietal spindle density maturation was similar, we restricted our analyses to frontal and central electrode sites. As stated above, overnight memory change was not correlated between INI and FUP. Therefore, we analyzed both time points separately. Based on the rationale that sleep‐dependent declarative memory consolidation is often shown by less forgetting rather than a real performance improvement, we divided the participants depending on their overnight memory change into memory maintainers (i.e. optimal performance; delayed recall − immediate recall ≥ 0) and memory decliners (delayed recall − immediate recall < 0; for descriptive data see Tables 2 and 3). Spindle density was investigated using a mixed ANOVA with the factors Night (baseline, experimental night), Memory Group (decliners, maintainers) and Spindle Type (slow, fast). Only significant main and interaction effects are reported in the main text, but a complete summary can be found in Table S3. At both recording times, decliners and maintainers did not differ significantly for possibly confounding variables, like IQ, age or puberty stage (all *p* > 0.05). At INI, however, memory decliners had a shorter total sleep time in the baseline night than memory maintainers (*t*(32) = −2.15, *p* = 0.03, *d* = −0.75). Furthermore, memory maintainers performed significantly better at the immediate recall than decliners at FUP (*t*(32) = −2.31, *p* = 0.04, *d* = −0.79). No other differences were found between the groups at both time points.

**Table 2:** Possible confounding variables for memory decliners and maintainers at initial recordings

	DECLINER (−)	MAINTAINER(+)	*p* ‐ values
N	20	14	
Overnight memory change	−6.35 ± 5.61	2.43 ± 2.03,	< .001*
Immediate recall	55.35 ± 16.66	48.14 ± 26.20	.33
IQ	105.5 ± 6.21	109.29 ± 8.03	.13
Age	9.50 ± 0.83	9.36 ± 0.74	.61
Puberty Score	1.65 ± 0.88	1.43 ± 0.85	.47

Sleep architecture	BASELINE	EXPERIMENTAL	BASELINE	EXPERIMENTAL	BASE_(‐)_*BASE_(+)_	EXP_(‐)_*EXP_(+)_
TIB(min)	573.90 ± 37.19	588.25 ± 40.20	599.89 ± 43.85	589.14 ± 36.73	.07	.95
SOL to N2(min)	19.25 ± 11.07	23.25 ± 20.39	20.61 ± 9.89	24.68 ± 7.41	.72	.80
WASO(min)	14.48 ± 16.64	6.70 ± 6.24	11.54 ± 9.93	12.18 ± 12.04	.56	.09
TST(min)	542.65 ± 39.16	563.55 ± 32.78	573.79 ± 44.64	560.00 ± 39.22	.04*	.78
EFF(%)	94.59 ± 3.47	95.80 ± 3.04	95.76 ± 2.11	95.03 ± 2.12	.27	.42
N1(%)	3.09 ± 3.10	2.42 ± 1.85	2.69 ± 1.60	2.91 ± 1.85	.66	.45
N2(%)	45.20 ± 6.83	44.56 ± 9.03	43.85 ± 8.47	43.42 ± 9.03	.61	.69
N3(%)	25.65 ± 10.1	25.11 ± 10.72	27.5 ± 9.40	26.12 ± 10.72	.59	.76
R(%)	26.06 ± 7.03	27.91 ± 7.91	25.96 ± 4.8	27.55 ± 7.59	.96	.89

Note

TIB, time in bed; SOL to N2, sleep onset latency; WASO, wake after sleep onset; TST, total sleep time; EFF, sleep efficiency.

Asterisks indicate a significant difference at the .05 level

**Table 3:** Possible confounding variables for memory decliners and maintainers at follow‐up recordings

	DECLINER (−)	MAINTAINER (+)	*p* ‐ values
N	17	17	
Overnight memory change	−3.71 ± 2.01	2.32 ± 1.68	< .001*
Immediate recall	65.51 ± 25.14	82.54 ± 17.07	.03*
IQ	106.76 ± 7.72	108.59 ± 9.75	.55
Age	15.94 ± 0.90	16.00 ± 0.87	.85
Puberty Score	4.18 ± 0.64	4.41 ± 0.62	.28

Sleep architecture	BASELINE	EXPERIMENTAL	BASELINE	EXPERIMENTAL	BASE_(‐)_*BASE_(+)_	EXP_(‐)_*EXP_(+)_
TIB(min)	507.06 ± 67.37	495.12 ± 41.32	481.85 ± 47.64	493.09 ± 54.75	.22	.90
SOL to N2(min)	23.32 ± 27.80	15.26 ± 9.12	16.59 ± 10.54	18.35 ± 16.84	.36	.51
WASO(min)	21.91 ± 23.89	10.88 ± 7.67	9.85 ± 6.58	9.18 ± 5.89	.05	.47
TST(min)	479.88 ± 76.76	475.12 ± 41.52	463.76 ± 45.47	474.53 ± 49.03	.46	.97
EFF(%)	94.50 ± 5.87	96.11 ± 2.65	96.28 ± 2.15	96.39 ± 1.81	.25	.72
N1(%)	9.96 ± 5.08	9.3 ± 4.33	8.32 ± 3.00	8.30 ± 5.09	.26	.54
N2(%)	42.18 ± 8.35	41.04 ± 8.25	41.11 ± 5.71	41.30 ± 4.83	.66	.91
N3(%)	23.86 ± 12.78	24.20 ± 12.19	26.17 ± 5.92	25.09 ± 6.04	.50	.79
R(%)	24.00 ± 7.11	25.46 ± 5.40	24.41 ± 3.52	25.31 ± 5.04	.83	.94

Note

TIB, time in bed; SOL to N2, sleep onset latency; WASO, wake after sleep onset; TST, total sleep time; EFF, sleep efficiency.

Asterisks indicate a significant difference at the .05 level

#### 3.4.1 Initial recordings (INI)

Neither absolute values of fast nor slow spindle density were correlated to overnight memory change. However, central slow spindle density was related to memory performance before (r_s_(34) = 0.43, *p* = 0.011) and after sleep (r_s_(34) = 0.37, *p* = 0.031).

**Figure 4:**
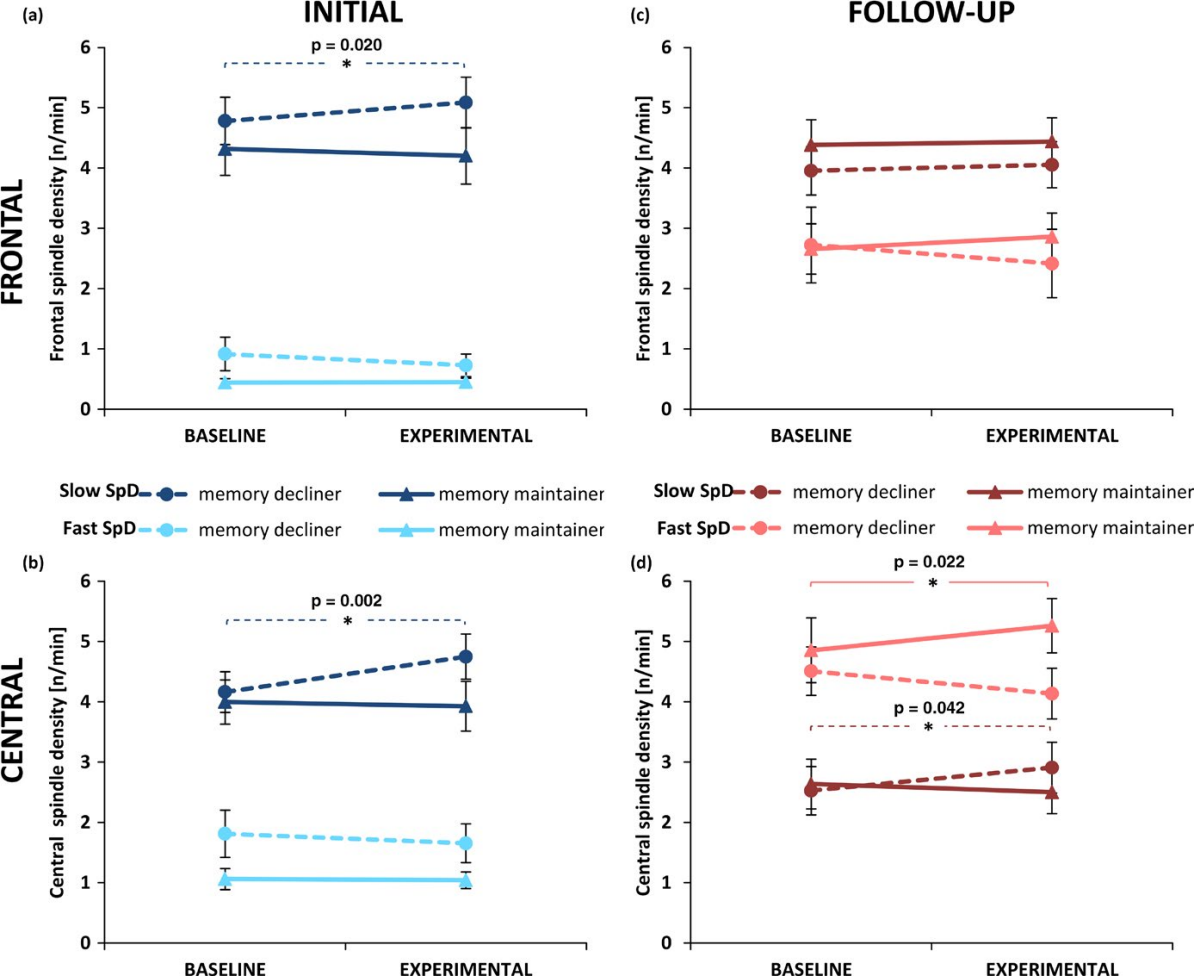
Means and standard error of slow (dark colours) and fast spindle density (bright colours) for memory decliners (dashed line) and maintainers at initial recording (left column, blue) and follow‐up recording (right column, red) at frontal (upper row) and central electrode sites (lower row) during baseline and experimental night. Memory decliner increased slow spindle density from baseline to experimental night at both electrode sites at initial recordings (frontal: p = 0.020; central: p = 0.002). At follow‐up recordings, memory maintainer increased fast spindle density from baseline to experimental night (p = 0.022), whereas memory decliner increased slow spindle density at central electrode sites (p = 0.042). *Note*: Group composition of memory decliner and memory maintainer is different between initial and follow‐up recordings

At frontal derivations (Figure 4a), slow spindle density was overall higher than fast spindle density (*F*(1, 32) = 196.91, *p* < 0.001, p.eta² = 0.86). Frontal spindle density differed depending on Night, Memory Group and Spindle Type (*F*(1, 32) = 4.74, *p* = 0.037, p.eta² = 0.13). Post‐hoc tests did not reveal differences between memory decliners and maintainers (all *p* > 0.111). However, memory decliners significantly increased their slow spindle density (*t*(19) = −2.54, *p* = 0.020†, *d* = −0.16) and showed a trend to decrease their fast spindle density from baseline to experimental night (*t*(19) = 1.83, *p* = 0.082, *d* = 0.09). Memory maintainers did not show significant changes in slow or fast spindle density from baseline to experimental night (both *p* > 0.635). Frontal slow spindle density enhancement from baseline to experimental night was negatively related to overnight memory change (r_s_(34) = −0.37, *p* = 0.032) even when controlling for immediate recall performance (r_p_(31) = −0.35, *p* = 0.046). Frontal fast spindle density did not show any relationship to overnight memory change.

At central derivations (Figure 4b), slow spindle density was overall higher than fast spindle density (*F*(1, 32) = 73.96, *p* < 0.001, p.eta² = 0.70). Independent of memory group, slow spindle density was higher during the experimental night than during the baseline night (*F*(1, 32) = 5.34, *p* = 0.028, p.eta² = 0.14). Central spindle density differed depending on Night, Memory Group and Spindle Type (*F*(1, 32) = 7.00, *p* = 0.013, p.eta² = 0.18). Post‐hoc tests did not reveal differences between memory decliners and maintainers (all *p* > 0.092). Memory decliners significantly increased central slow spindle density (*t*(19) = −3.65, *p* = 0.002, *d* = 0.37) from baseline to experimental night. Memory maintainers did not show any changes in slow or fast spindle density from baseline to experimental night (all *p* > 0.694). Similar to frontal derivations, central slow spindle density enhancement from baseline to experimental night was negatively related to overnight memory change (r_s_(34) = −0.45, *p* = 0.008) even when controlling for immediate recall performance (r_p_(31) = −0.44, *p* = 0.011). Fast spindle density did not show any relationship to overnight memory change.

#### 3.4.2 Follow‐up recordings (FUP)

At frontal sites (Figure 4c), slow spindle density was higher than fast spindle density (*F*(1, 32) = 12.20, *p* = 0.001, p.eta² = 0.28). In addition, frontal spindle density showed a trend to differ depending on Night, Memory Group and Spindle Type (*F*(1, 32) = 2.81, *p* = 0.104, p.eta² = 0.08). In contrast to INI, slow spindle density changes from baseline to experimental night were unrelated to overnight memory change (r_s_(34) = 0.17, *p* = 0.333) at FUP. However, higher increases in fast spindle density from baseline to experimental night resulted in a positive overnight memory change (r_s_(34) = 0.37, *p* = 0.030). This relationship still persisted when controlling for immediate recall performance (r_p_(31) = 0.34, *p* = 0.050).

At central derivations (Figure 4d), fast spindle density was higher than slow spindle density (*F*(1, 32) = 10.63, *p* = 0.003, p.eta² = 0.25). However, spindle density changed in relation to Night, Memory Group and Spindle Type (*F*(1, 32) = 8.40, *p* = 0.007, p.eta² = 0.21). Post‐hoc tests between the groups were not significant (all *p* > 0.205), but tests within the groups revealed that memory decliners significantly increased their slow spindle density (*t*(16) = −2.22, *p* = 0.042†, *d* = −0.23), whereas memory maintainers significantly increased their fast spindle density from baseline to experimental night (*t*(16) = −2.54, *p* = 0.022†, *d* = −0.19). Central slow spindle density increases from baseline to experimental night showed a trend for a negative correlation with overnight memory change (r_s_(34) = −0.32, *p* = 0.074). Participants with a higher increase in fast spindle density showed a positive overnight memory change (r_s_(34) = 0.36, *p* = 0.036), even when controlling for immediate recall performance (r_p_(31) = 0.41, *p* = 0.018).

Together, these results indicate that fast spindle density enhancement is beneficial for sleep‐dependent memory consolidation. The negative effect of slow spindle density on memory consolidation might be striking at the first glance; however analyses of spindle frequency (see Table S4 and Figure S5) suggest that the underlying mechanism could be a change in spindle frequency. Spindle frequency increases were positively associated with overnight memory change, whereas spindle frequency decreases were negatively associated with overnight memory changes.

### 3.5 Developmental central fast sleep spindle changes related to memory consolidation

**Figure 5:**
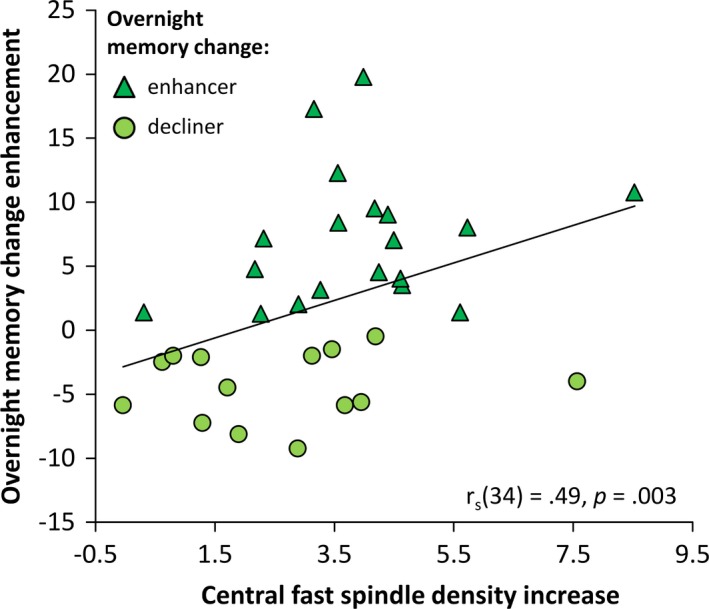
Spearman correlations between the development of overnight memory change enhancement and central fast spindle density development. Higher increase in central fast spindle density in the experimental night from initial to follow‐up was related to enhanced overnight memory change relative to initial recordings. Participants who enhance overnight memory change from initial to follow‐up are indicated by triangles, those who decline by circles

In a next step we further investigated whether developmental spindle density changes can explain differences in overnight memory changes. We found that an enhancement in overnight memory change between INI and FUP was related to decreased central slow spindle density (r_s_(34) = −0.33, *p* = 0.056) and especially to increased central fast spindle density between INI and FUP during the experimental nights (r_s_(34) = 0.49, *p* = 0.003; Figure 5). A comparison of correlations showed that enhancement in overnight memory change correlated significantly higher with fast spindle density development than with slow spindle density development (z = 2.75, *p* = 0.003). As age and pubertal development stage are known to influence spindle density, we computed partial correlations controlling for age and pubertal stage differences. These correlations revealed a stable relationship between fast spindle density development and the enhancement of overnight memory change (r_p_(30) = 0.57, *p* < 0.001). When comparing participants who relatively enhanced their overnight memory change from INI to FUP with those who relatively declined, an independent *t* test showed that enhancers had a significantly higher increase in fast spindle density across the seven years in the experimental night than decliners (*t*(32) = −2.17, *p* = 0.037, *d* = −0.75). These findings indicate that participants with a higher increase in fast spindle density showed stronger sleep benefits on memory consolidation and suggest that the development of fast sleep spindles could be critical for sleep dependent memory consolidation.

### 3.6 Developmental frontal slow sleep spindle changes related to cognitive abilities

**Figure 6:**
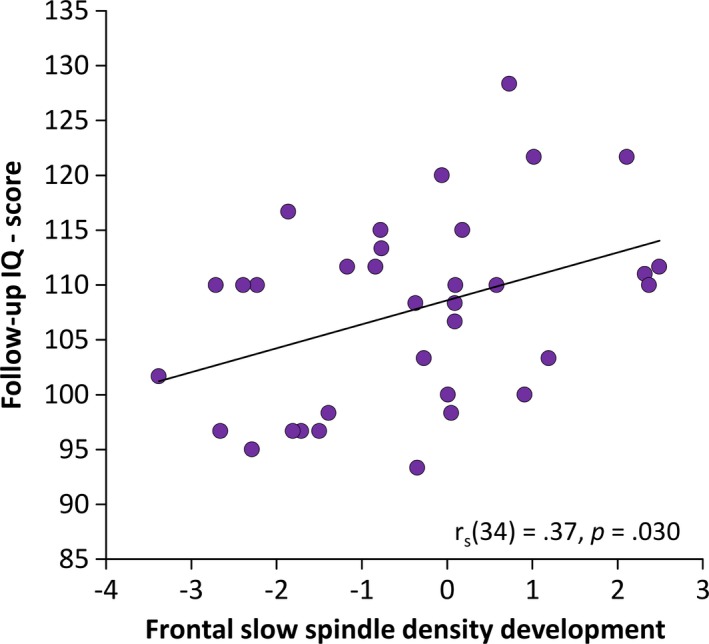
Spearman correlations between the mean IQ score at follow‐up recordings and slow spindle density development on frontal sites with linear trend line. An increase in frontal slow spindle density was linked to higher cognitive abilities (IQ)

Participants showed an intelligence score of 107.06 ± 7.15 at INI and 107.68 ± 8.71 at FUP. Intelligence scores were highly correlated between the two recordings (r_s_(34) = 0.73, *p* < 0.001). We found no correlations between absolute spindle densities (fast, slow) and intelligence scores at both recording times. Likewise, frontal fast spindle density development was not correlated to intelligence scores at FUP (r_s_(34) = 0.04, *p* = 0.805). However, participants who increased frontal slow spindle density during the baseline night from INI to FUP had higher intelligence scores at FUP (r_s_(34) = 0.37, *p* = 0.030; Figure 6). This relationship still persisted when controlling for age and pubertal stage differences (r_p_(30) = 0.37, *p* = 0.038). A comparison of correlations showed that intelligence scores showed a trend to correlate more highly with slow spindle density development than with fast spindle density development (*z* = 1.51, *p* = 0.066). The correlation between frontal slow spindle density development and cognitive abilities was also present for the experimental night but failed to reach significance (r_s_(34) = 0.32, *p* = 0.062). Overall, this suggests that slow spindle development might be involved in the development of cognitive functions.

## 4 DISCUSSION

The aim of this longitudinal study was to investigate the development of sleep spindles and their significance for sleep‐dependent declarative memory consolidation as well as cognitive abilities.

As hypothesized, memory performance and sleep‐dependent memory consolidation improved during adolescence. Furthermore, memory was more strongly consolidated during sleep in comparison to wakefulness during adolescence. We found that fast spindles were not only developing, but also related to sleep‐dependent memory consolidation enhancement throughout adolescence. While slow spindle development was not as pronounced as fast spindle development, it was still related to general cognitive abilities.

### 4.1 Declarative memory performance and memory consolidation

On a behavioral level, we found that recall performance increases from childhood to adolescence, even though we increased the difficulty of the word pair task (Figure 2a). This development is most likely due to the enhanced mental skills of adolescents. For example, adolescents are better at using memory strategies, manipulating encoded memories, and forming semantic connections because of their superior world knowledge and experiences (Bjorklund & Schneider, 1996; Cowan, Nugent, Elliott, Ponomarev, & Saults, 1999; Crone, Wendelken, Donohue, van Leijenhorst, & Bunge, 2006).

With respect to the effect of sleep on memory consolidation, we found that declarative memory declines during childhood after sleep (Figure 2a). In contrast, during adolescence participants did benefit from sleep as opposed to a period of wakefulness, with sleep maintaining word pair memory and wakefulness promoting forgetting (Figure 2b). This result is in line with previous studies investigating young adults, and further underscores the beneficial effect of sleep on memory consolidation (Diekelmann & Born, 2010; Walker & Stickgold, 2004). It might seem like a marginal effect that declarative memory was only maintained but not increased after sleep. However, declarative memory is more prone to decay or interference than procedural memory, which is why ‘successful’ sleep‐dependent declarative memory consolidation is in general viewed as less forgetting after sleep compared to after wakefulness (Diekelmann, Wilhelm, & Born, 2009).

Our earlier findings revealed that children decreased their declarative memory performance over sleep (Hoedlmoser et al., 2014). This is still the case for the reanalyzed subset of participants in the present study (Figure 2a), a result that contradicts earlier studies (Backhaus et al., 2008; Urbain, Di Vincenzo, Peigneux, & Van Bogaert, 2011; Wilhelm et al., 2008). Possible reasons for the absence of a positive sleep effect on memory consolidation in children have already been discussed in more detail in our previous publication (Hoedlmoser et al., 2014). In short, the memory task could have been too difficult for our participants during childhood and, because sleep‐dependent memory consolidation is influenced by how strongly a memory is encoded, a task that was too difficult could therefore have prevented sleep dependent memory benefits (Drosopoulos, Schulze, Fischer, & Born, 2007). Because recall performance was overall worse during childhood, the difference in encoding strength could also explain why declarative memory declined overnight during childhood as opposed to during adolescence. Furthermore, it has to be noted that in the current study we did not conduct an additional wake condition during childhood. This limitation makes it impossible to give a sound statement about whether there were sleep‐dependent memory consolidation benefits during childhood or not.

Connecting both developmental stages, we found a strong relationship of recall performance as well as general cognitive abilities between childhood and adolescence. This result is not surprising given that general cognitive abilities are considered a trait‐like aspect which, by definition, should remain stable over time. But in contrast to this finding, there was no relationship of the overnight memory change between childhood and adolescence, which in turn might suggest that sleep‐dependent memory consolidation must be considered a state‐like aspect. However, in the following paragraphs we will discuss the development of sleep spindles and whether this process could also account for the missing link of sleep‐dependent memory consolidation between childhood and adolescence.

### 4.2 Maturational changes of sleep spindles

In line with previous reports, we found that, overall, both spindle density (Figure 3) and frequency (Figure S4) increase during adolescence (Campbell & Feinberg, 2016; Purcell et al., 2017; Scholle et al., 2007; Shinomiya et al., 1999; Tarokh, Carskadon, & Achermann, 2011). Higher spindle density is associated with increased white matter diffusion along axons (Piantoni et al., 2013). Likewise, it is proposed that higher EEG frequencies are produced by more extensive myelination in cortical networks, resulting in enhanced transmission speed along axons in the form of action potentials (Nunez, 2000). Given the thalamo‐cortical origin of sleep spindles, it seems plausible that the increase in spindle density and frequency represents enhanced myelination in thalamo‐cortical circuits, giving rise to a more mature and effective network (Steriade et al., 1985; Tarokh et al., 2011).

As hypothesized, when distinguishing between slow and fast spindles, we showed that slow spindle density was dominant at all electrode sites during childhood (Figure 3), whereas fast spindle density only became dominant at centro‐parietal sites across adolescence. As a result, the topographical pattern of slow spindles predominating frontal areas while fast spindles are more pronounced in centro‐partial areas resembled the earlier reported typical spindle topography only during adolescence (Anderer et al., 2001; Zeitlhofer et al., 1997). Therefore we conclude that the mature spindle topography develops throughout puberty.

Overall, slow spindle density decreased whereas fast spindle density increased, similar to the findings that slow spindle power decreases, but fast spindle power increases at central electrodes (Campbell & Feinberg, 2016). In addition our data showed that only at frontal electrodes the dominance of slow spindles over fast spindles persisted across the seven years, a finding that might indicate that frontal spindles mature earlier than centro‐parietal spindles (Purcell et al., 2017). These different developmental time courses of slow and fast spindles further support the notion of two different spindle types with presumably distinct functions (Schabus et al., 2007; Zeitlhofer et al., 1997).

It has to be noted that our sample had a bias towards female participants. Given that males and females show differences in brain maturation from childhood to adolescence, the sample composition has to be considered as a limitation of our study. Furthermore, with spindle frequency also increasing through adolescence, it is challenging to identify the process that drives slow and fast spindle differentiation. One explanation could be that fast spindles already exist in childhood but are still nested in the slow spindle frequency range, and only through the frequency acceleration during adolescence are they detected as such. An alternative explanation could be that spindle generators simply start to generate more fast spindles, which would also increase overall spindle frequency. In this vein, it still needs to be investigated whether a fixed ad hoc frequency boundary or a more continuous frequency distinction is better for describing slow and fast spindle characteristics.

### 4.3 Memory consolidation and sleep spindles

As hypothesized, spindle density changed from baseline to experimental night depending on whether memory was maintained or declined after sleep (Figure 4). During childhood and adolescence, increases in slow spindle density were associated with a memory decline. In contrast, increases in fast spindle density were only associated with memory maintenance during adolescence. The overall reduced spindle frequency and lower density of fast spindles during childhood might have circumvented the detection of a similar effect during childhood (Figures 3 and S4).

The relationship between slow spindle density and overnight memory decline could be explained by sleep spindle frequency analyses. We found that sleep spindle frequency acceleration benefited memory consolidation whereas sleep spindle frequency deceleration interfered with memory consolidation (Figure S5). Therefore we argue that the negative effect of the slow spindle density increase from baseline to experimental night on memory consolidation might not be the effect of the slow spindle type, but rather a result of a frequency deceleration of fast spindles, causing fast spindles to pass the predefined boundary (i.e. 13 Hz in our study). However, as to why spindle frequency deceleration or slow spindles would interfere with memory consolidation is currently unclear.

The beneficial impact of fast spindle density increases from baseline to experimental night on memory consolidation is in line with Schabus et al. (2004), who found sleep‐dependent memory increases only for subjects with spindle activity increments in the experimental night as compared to a control night. More precisely, the increase in fast spindle activity, in particular, resulted in sleep‐dependent memory improvements, whereas a decrease in fast spindle activity was shown in memory non‐improvers (Schabus et al., 2008).

Evidence of fast spindles being beneficial for sleep‐dependent declarative memory consolidation is accumulating (Cox, Hofman, de Boer, & Talamini, 2014; Groch, Schreiner, Rasch, Huber, & Wilhelm, 2017; Mölle, Bergmann, Marshall, & Born, 2011; Tamminen, Payne, Stickgold, Wamsley, & Gaskell, 2010). In contrast to slow spindles, fast spindles are related to hemodynamic hippocampal activation and hippocampal sharp wave ripple events (Clemens et al., 2011; Schabus et al., 2007). The hippocampus plays a crucial role in sleep‐dependent memory consolidation as it has been shown to be involved in neuronal replay of previously acquired memory traces during wakefulness (Ji & Wilson, 2007). Through this replay, which is associated with hippocampal sharp wave ripple events, memory is transferred from hippocampal short‐term memory systems to neocortical long‐term memory systems (Buzsaki, 1996; Ji & Wilson, 2007). In our study, we speculate that memory was maintained due to increased memory replay, a process that is represented by increases in fast spindle density. Taken together our results suggest that fast spindles rather than slow spindles are beneficial for sleep‐dependent memory consolidation.

As a key finding of our longitudinal data analyses, we report for the first time that fast spindle development is related to sleep‐dependent memory consolidation. Higher increases in fast spindle density from childhood to adolescence predicted the improvement of sleep‐dependent memory consolidation across this time period (Figure 5). This connection remained stable when controlling for age and pubertal stage differences, indicating that the improvement in sleep‐dependent memory consolidation is not just related to aging but is closely tied to fast spindle development. Therefore, this finding implies that differences in memory consolidation between children and adolescents could be explained by underdeveloped fast spindles.

It has been earlier hypothesized that children show inferior sleep‐dependent memory consolidation compared to adults because they lack the already established memory representations in which new memories are integrated (Wilhelm, Metzkow‐Meszaros, Knapp, & Born, 2012). Sleep spindles seem to be especially crucial for the integration of new memories in already existing long‐term memory, emphasizing a dependency of prior knowledge (Tamminen et al., 2010). Beyond that, cued memory reactivation during sleep was only beneficial for memories related to already established knowledge, which was accompanied by an increase in fast spindle activity (Groch et al., 2017). Therefore, the relationship between fast spindle development and sleep‐dependent memory consolidation change could, to some extent, be attributed to more elaborate memory representations during adolescence. Nonetheless, our results speak for a crucial role of fast sleep spindles and their development for sleep‐dependent memory consolidation.

### 4.4 Sleep spindles and cognitive abilities

With the longitudinal study design, we were able to connect developmental sleep spindle changes with cognitive abilities. We found that the higher the developmental increase in frontal slow spindle density, the higher the general cognitive abilities (Figure 6), a result that further contributes to the picture of sleep spindles as a physiological marker of intelligence (Fogel & Smith, 2011; Schabus et al., 2006).

At first glance it might seem puzzling that slow spindle density is not beneficial for sleep‐dependent memory consolidation (Figure 4), but promote better cognitive functioning. However, while it is true that higher intelligence positively impacts the learning of new material, it does not impact sleep‐dependent memory consolidation to a considerable extent (Tucker & Fishbein, 2009). Corroborating this idea, we found that intelligence scores only positively correlated with immediate recall performance but not with overnight memory change. If we consider sleep‐dependent memory consolidation an endogenous, unconscious process and acquiring knowledge an active, conscious process, it is not contradictory that slow sleep spindles are not associated with sleep‐dependent memory consolidation but instead with cognitive functioning.

But what does this association mean on a neural level? Slow spindles are associated with cortico‐cortical network couplings and are further proposed to reflect thalamic projections to frontal cortices (Andrillon et al., 2011; Doran, 2003). Frontal cortices are thought to be the domain of executive functions that support intelligent behavior (Gray & Thompson, 2004). However, executive functions within frontal cortices are still under development until late adolescence (Luna, Garver, Urban, Lazar, & Sweeney, 2004; Scherf, Sweeney, & Luna, 2006). Therefore changes in slow spindle density might reflect the development of executive functions in the frontal cortex.

That brain development trajectories rather than absolute values (in that case cortical thickness) can predict intelligence was already shown in a neuroimaging study (Shaw et al., 2006). More intelligent children showed higher cortical thickness at the beginning of puberty and a more prominent subsequent cortical thinning and thus a very plastic (i.e. modifiable) cortex during puberty. In relation to spindles, albeit speculating, the higher cortical thickness might be represented by the elevated slow spindle activity in highly gifted children as we reported earlier (Hoedlmoser et al., 2014), while the present slow spindle density development could reflect differences in cortical thinning, which results in higher cognitive abilities. This might indicate that the relationship of sleep spindles and intelligence, a relationship that could be considered trait‐like, is a dynamic one across aging. This is further corroborated by numerous different sleep spindle parameters that have been linked to cognitive abilities in different age groups. Given the extensive changes of spindles across aging, it seems unsurprising that there is no common ground in terms of spindle parameters related to cognitive abilities.

### 4.5 Conclusion

Our results demonstrate that the typical mature spindle topography with slow spindles dominating frontal areas and fast spindles centro‐parietal areas develops throughout adolescence. Fast spindles seem to be crucial for sleep‐dependent memory consolidation as their maturation is related to more efficient sleep‐dependent memory consolidation. In contrast, slow spindle development appears to contribute to the generation of frontal brain networks relevant for efficient cognitive processing.
