# Supplementary material for: Developmental changes of sleep spindles and their impact on sleep‐dependent memory consolidation and general cognitive abilities: A longitudinal approach
Source: Dev Sci. 2018 Sep 5;22(1):e12706. doi: 10.1111/desc.12706 (PMC6492121; doi:10.1111/desc.12706)
Supplement: Supplementary file 1 [file DESC-22-na-s001.docx]

**SUPPLEMENTAL MATERIAL**

**Developmental changes of sleep spindles and their impact on sleep-dependent memory consolidation and general cognitive abilities – a longitudinal approach**

Hahn, M. ^1^, Joechner, A.^1^, Roell J.^1^, Schabus, M. ^1^, Heib, D.P.J.^1^, Gruber, G.^2,3^,
Peigneux, P.^4^ & Hoedlmoser, K.^1*^

1 Laboratory for Sleep, Cognition and Consciousness Research, Department of Psychology, Centre for Cognitive Neuroscience, University of Salzburg, Salzburg, Austria

2 Department of Psychiatry and Psychotherapy, Medical University of Vienna, Vienna, Austria

3 The Siesta Group, Vienna, Austria

4 UR2NF - Neuropsychology and Functional Neuroimaging Research Unit affiliated at CRCN - Centre de Recherches en Cognition et Neurosciences and UNI - ULB Neurosciences Institute, Université Libre de Bruxelles, Bruxelles, Belgium

*Corresponding author information:

Kerstin Hoedlmoser, PhD

Address: Hellbrunnerstrasse 34, 5020 Salzburg, Austria

Tel.: 0043 662 8044 5143

E-Mail: Kerstin.Hoedlmoser@sbg.ac.at

**Supplementary tables**

**Table S1** *Summary of the effects of the spindle density maturation ANOVAs during baseline and experimental night*

| Effect | BASELINE | EXPERIMENTAL |
| --- | --- | --- |
| Maturation | *F*(1, 33) = 33.12, *p* < .001, p.eta² = .50 | *F*(1, 33) = 35.63, *p* < .001, p.eta² = .52 |
| Electrode | *F*(1.58, 51.37) = 7.30, *p* = .008, p.eta² = .16 | *F*(1.61, 53.03) = 7.30, *p* = .003, p.eta² = .18 |
| Spindle Type | *F*(1, 33) = 6.19, *p* = .018, p.eta² = .16 | *F*(1, 33) = 11.57, *p* = .002, p.eta² = .25 |
| Maturation*Electrode | *F*(1.55, 51.29) = 1.08, *p* = .335, p.eta² = .03 | *F*(1.33, 43.89) = 0.27, *p* = .671, p.eta² = .01 |
| Maturation*Spindle Type | *F*(1, 33) = 61.99, *p* < .001, p.eta² = .65 | *F*(1, 33) = 68.27, *p* < .001, p.eta² = .67 |
| Electrode*Spindle Type | *F*(1.15, 37.82) = 53.61, *p* < .001, p.eta² = .62 | *F*(1.12, 37.24) = 45.63, *p* < .001, p.eta² = .58 |
| Maturation*Electrode*Spindle Type | *F*(1.25, 41.25) = 32.45, *p* < .001, p.eta² = .50 | *F*(1.29, 42.43) = 39.21, *p* < .001, p.eta² = .54 |

**Table S2** *Summary of the effects of the spindle frequency maturation ANOVAs during baseline and experimental night*

| Effect | BASELINE | EXPERIMENTAL |
| --- | --- | --- |
| Maturation | *F*(1, 33) = 303.43, *p* < .001, p.eta² = .90 | *F*(1, 33) = 305.21, *p* < .001, p.eta² = .90 |
| Electrode | *F*(2, 66) = 90.18, *p* < .001, p.eta² = .74 | *F*(2, 66) = 103.23, *p* < .001, p.eta² = .76 |
| Maturation*Electrode | *F*(2, 66) = 28.51, *p* < .001, p.eta² = .46 | *F*(2, 66) = 21.58, *p* < .001, p.eta² = .40 |
|  |  |  |

**Table S3** *Summary of the effects of night and memory group on slow and fast spindle density at frontal and central electrodes during initial and follow-up recordings*

| Effect | INITIAL | | FOLLOW-UP | |
| --- | --- | --- | --- | --- |
|  | Frontal | Central | Frontal | Central |
| Night | *F*(1, 32) = .002, *p* = .961, p.eta² < .001 | *F*(1, 32) = 1.55, *p* = .223, p.eta² = .046 | *F*(1, 32) = .035, *p* = .852, p.eta² = .001 | *F*(1, 32) = 1.36, *p* = .253, p.eta² = .041 |
| Spindle Type | *F*(1, 32) = 196.91, *p* < .001, p.eta² = .860 | *F*(1, 32) = 73.96, *p* < .001, p.eta² = .698 | *F*(1, 32) = 12.20, *p* = .001, p.eta² = .276 | *F*(1, 32) = 10.63, *p* = .003, p.eta² = .249 |
| Memory Group | *F*(1, 32) = 1.92, *p* = .175, p.eta² = .057 | *F*(1, 32) = 2.71, *p* = .110, p.eta² = .078 | *F*(1, 32) = .415, *p* = .524, p.eta² = .013 | *F*(1, 32) = .557, *p* = .461, p.eta² = .017 |
| Night*Spindle Type | *F*(1, 32) =1.75, *p* = .196, p.eta² = .052 | *F*(1, 32) = 5.34, *p* = .028, p.eta² = .143 | *F*(1, 32) = .61, *p* = .442, p.eta² = .019 | *F*(1, 32) = .226, *p* = .638, p.eta² = .007 |
| Night*Memory Group | *F*(1, 32) = .71, *p* = .404, p.eta² = .022 | *F*(1, 32) = 3.59, *p* = .067, p.eta² = .101 | *F*(1, 32) = 2.99, *p* = .093, p.eta² = .086 | *F*(1, 32) = 1.17, *p* = .288, p.eta² = .035 |
| Spindle Type*Memory Group | *F*(1, 32) = .28, *p* = .600, p.eta² = .009 | *F*(1, 32) = .083, *p* = .776, p.eta² = .003 | *F*(1, 32) = 0.61, *p* = .806, p.eta² = .002 | *F*(1, 32) =.495, *p* = .487, p.eta² = .015 |
| Night*Spindle Type*Memory Group | *F*(1, 32) = 4.74, *p* = .037, p.eta² = .129 | *F*(1, 32) = 6.99, *p* = .013, p.eta² = .179 | *F*(1, 32) = 2.81, *p* = .104, p.eta² = .081 | *F*(1, 32) = 8.39, *p* = .007, p.eta² = .208 |

**Table S4** *Summary of the effects of night and memory group on spindle frequency at frontal and central electrodes during initial and follow-up recordings*

| Effect | INITIAL | | FOLLOW-UP | |
| --- | --- | --- | --- | --- |
|  | Frontal | Central | Frontal | Central |
| Night | *F*(1, 32) = 2.50, *p* = .124, p.eta² = .07 | *F*(1, 32) =.64, *p* = .430, p.eta² = .02 | *F*(1, 32) = .001, *p* = .977, p.eta² < .001 | *F*(1, 32) = .02, *p* = .882, p.eta² = .001 |
| Memory Group | *F*(1, 32) = .12, *p* = .731, p.eta² = .004 | *F*(1, 32) = .02, *p* = .895, p.eta² = .001 | *F*(1, 32) = 1.37, *p* = .251, p.eta² = .04 | *F*(1, 32) = 1.29, *p* = .265, p.eta² = .04 |
| Night*Memory Group | *F*(1, 32) = 9.02, *p* = .005, p.eta² = .22 | *F*(1, 32) = 7.31, *p* = .011, p.eta² = .19 | *F*(1, 32) = 9.75, *p* = .004, p.eta² = .23 | *F*(1, 32) = 11.21, *p* = .002, p.eta² = .26 |

**Supplementary Figures**

**
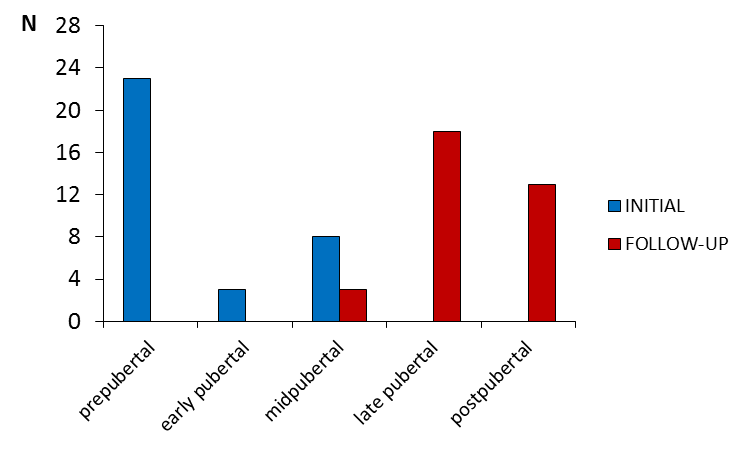
**

**Figure S1** *Distribution of pubertal development stage at initial and follow-up recordings.*

*
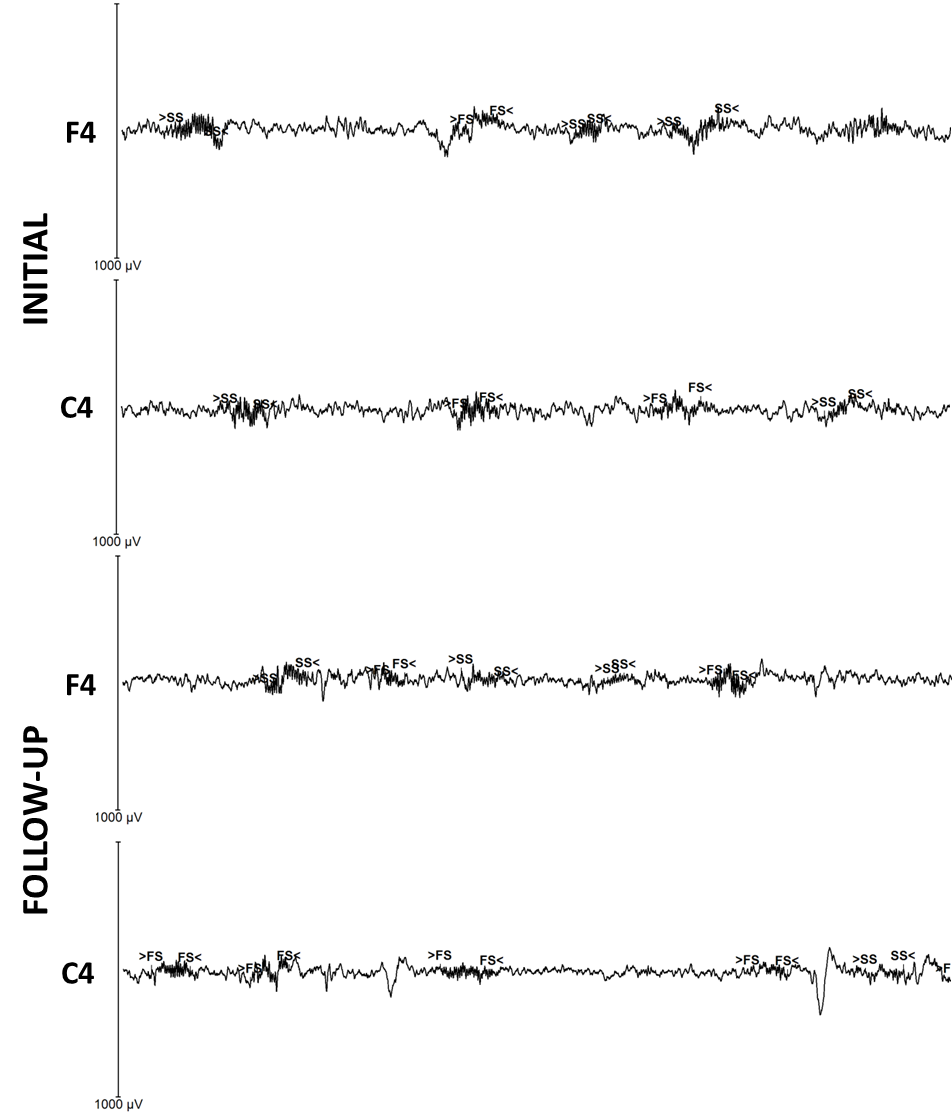
*

**Figure S2** *Exemplary detections by the automatic sleep spindle detection algorithm in unfiltered 30 s epochs for initial (upper row) and follow-up (lower row) recordings at electrode positions F4 and C4. Slow spindle events are indicated by SS, fast spindle events by FS. The start of a spindle event is marked by > whereas its termination is marked by <.*


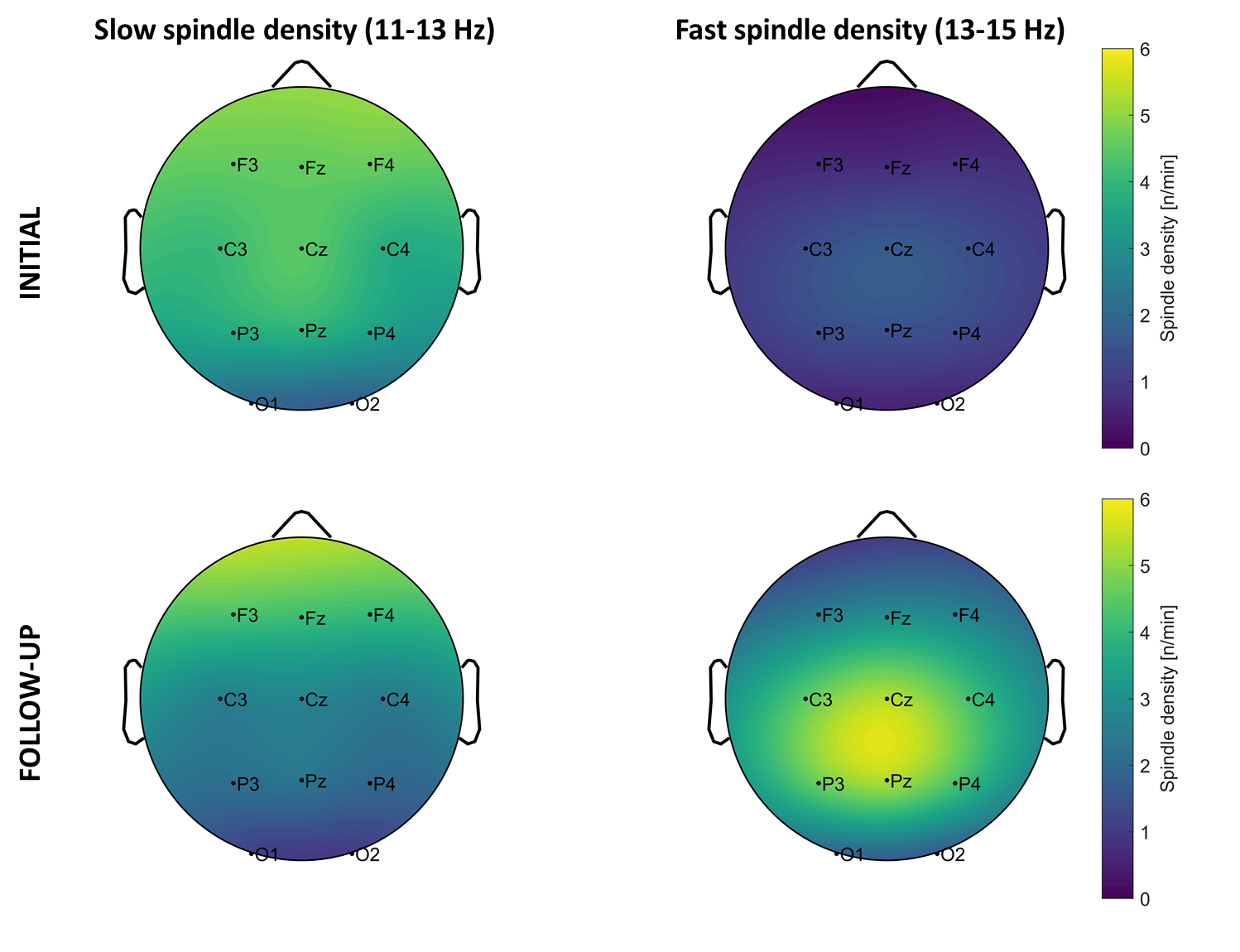


**Figure S3** *Topographical plots for mean spindle density at each electrode separated by slow (left column) and fast spindle density (right column) at initial (upper row) and follow-up recordings (lower row) during baseline nights. Bright colours indicate higher spindle density.* *Slow spindles were dominant during initial recordings. At follow-up slow spindles only remained dominant at frontal derivations, whereas fast spindles became dominant at centro-parietal derivations. Note that due to the extrapolation of topographical plots, colours that go beyond the measured electrodes do not contain any information.*


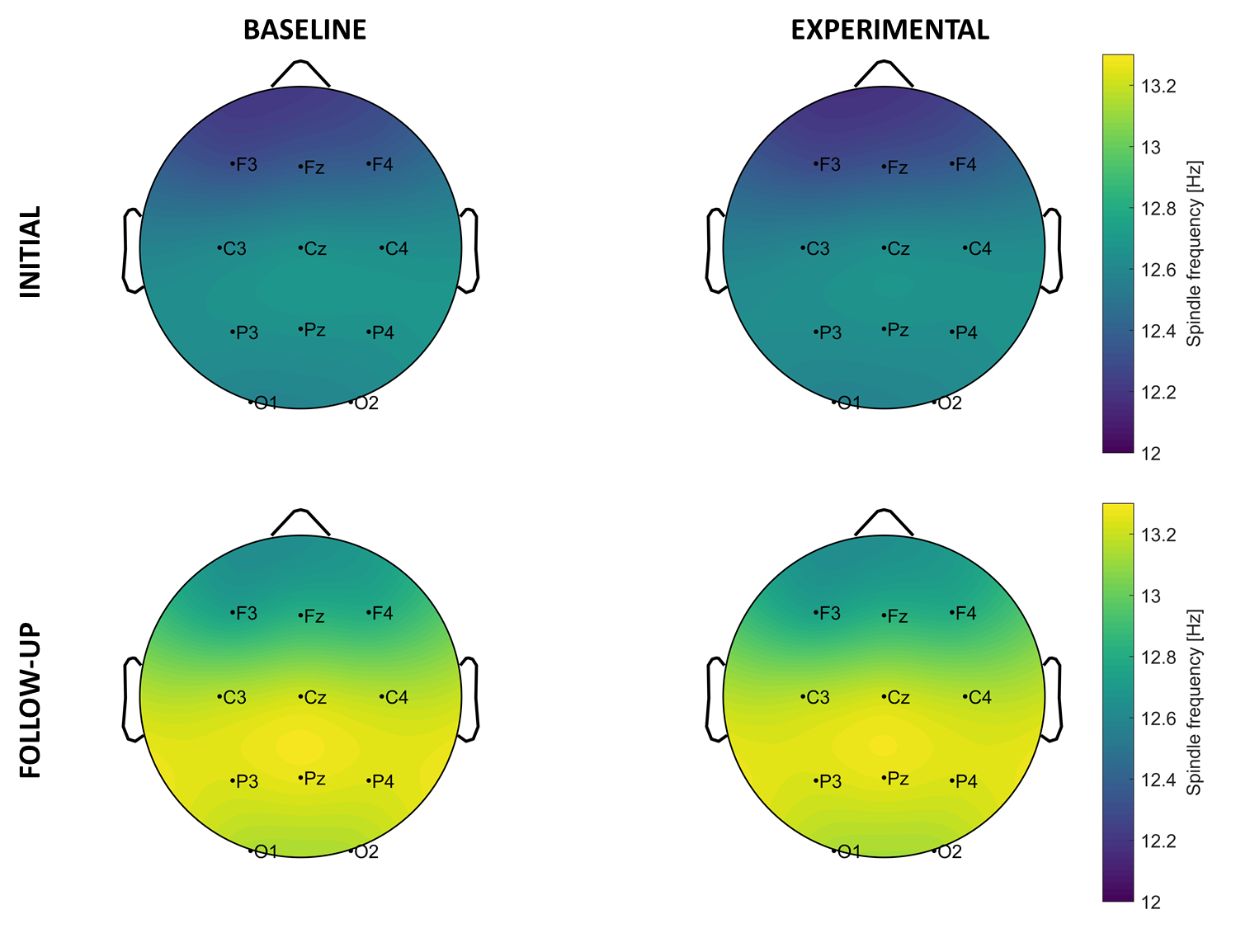


**Figure S4** *Topographical plots for mean spindle frequency (11-15 Hz) at each electrode at initial (upper row) and follow-up recordings (lower row) during baseline night (left column) and experimental night (right column). Bright colours indicate higher spindle frequency.* *Note that due to the extrapolation of topographical plots, colours that go beyond the measured electrodes do not contain any information.*


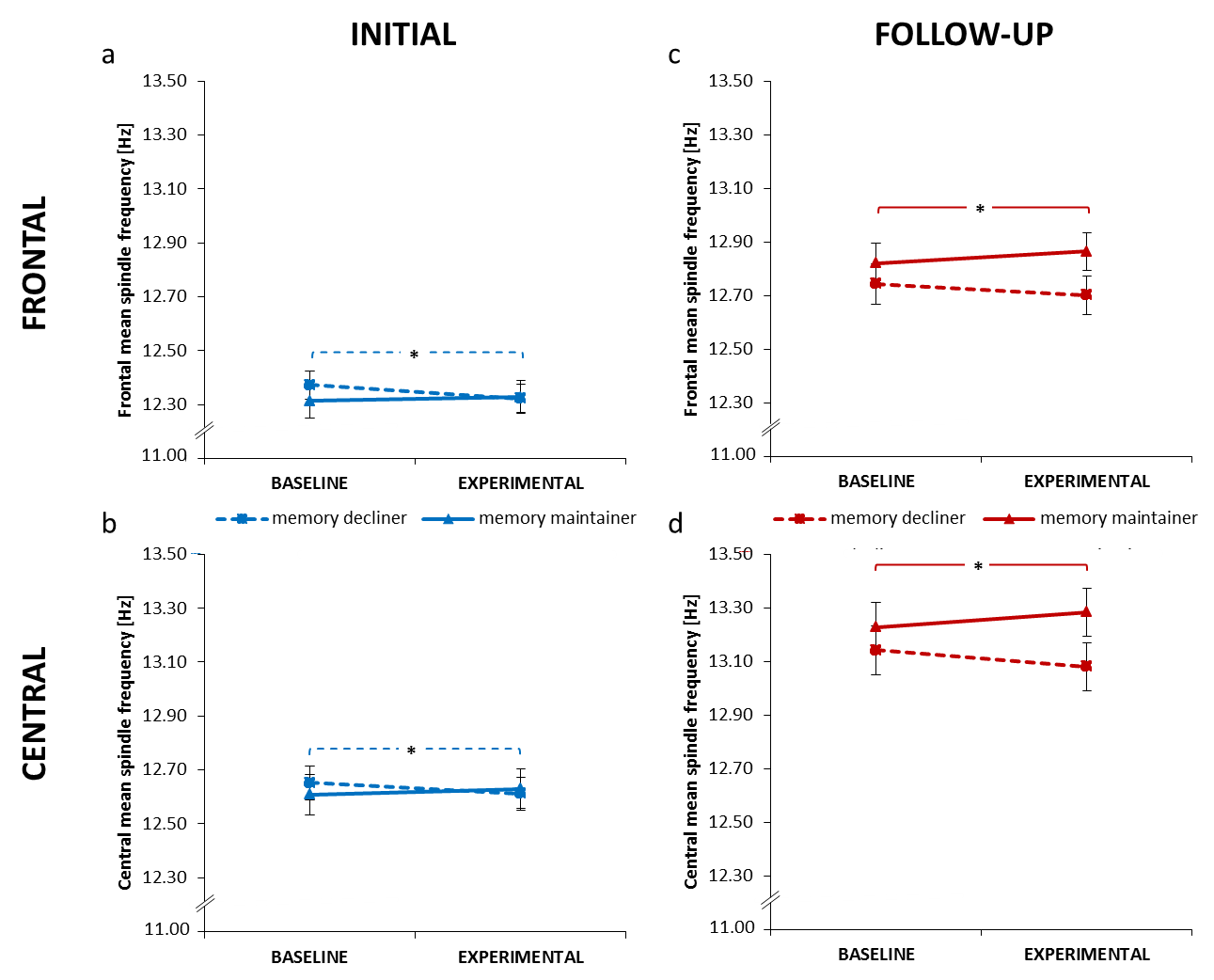


**p = 0.005**

**p = 0.006**

**p < 0.001**

**p = 0.003**

**Figure S5** *Means and standard error of spindle frequency for memory decliners (dashed line) and maintainers during baseline and experimental night. Initial recordings are indicated in blue, follow-up recordings in red. Results are presented at frontal (upper row) and central electrode sites (lower row). Memory decliners decelerate spindle frequency from baseline to experimental night at initial recordings, whereas memory maintainers accelerate spindle frequency from baseline to experimental night at follow-up recordings.*
